# BMSC-derived extracellular vesicles enhance osteosarcoma proliferation and metastasis via the circRNA-0010220/β-catenin pathway

**DOI:** 10.1038/s41419-026-08655-8

**Published:** 2026-03-25

**Authors:** Runsang Pan, Yujie Pan, Wanyuan Ruan, Hao Zheng, Guangfu Jiang, Jianyang Li, Xiaobin Tian, Li Sun

**Affiliations:** 1 Department of Orthopedics, GuiZhou Provincial People’s Hospital, Guiyang, China; 2 Department of Emergency, The Affiliated Hospital of Guizhou Medical University, Guiyang, China; 3 School of Clinical Medicine, Guizhou Medical University, Guiyang, China; 4 School of Forensic Medicine, Guizhou Medical University, Guiyang, China

**Keywords:** Bone cancer, Cancer stem cells

## Abstract

Osteosarcoma (OS) remains a challenging malignancy with a high propensity for metastasis and poor survival outcomes. Bone marrow mesenchymal stem cell-derived extracellular vesicles (BMSC-EVs) have emerged as key mediators in the tumor microenvironment, promoting OS progression. This study identifies a novel molecular axis centered on circRNA-0010220 within BMSC-EVs that drives OS aggressiveness. We demonstrate that BMSC-EVs are internalized by OS cells, enhancing their proliferation, migration, and invasion. High-throughput sequencing revealed circRNA-0010220 as the most significantly upregulated circRNA in EV-treated OS cells. Functional studies showed that circRNA-0010220 knockdown in BMSCs attenuated the oncogenic effects of their EVs both in vitro and in vivo. Mechanistically, circRNA-0010220 recruits the histone methyltransferase EZH2 to the CTNNBIP1 promoter, facilitating H3K27me3-mediated epigenetic silencing. The subsequent downregulation of CTNNBIP1 leads to activation of the Wnt/β-catenin signaling pathway. This cascade was consistently observed across gain-of-function and loss-of-function experiments, and pharmacologic inhibition of β-catenin reversed the pro-tumorigenic effects. Our findings elucidate a complete signaling axis from BMSC-EVs to Wnt/β-catenin activation via circRNA-0010220/EZH2/CTNNBIP1, providing new insights into the epigenetic regulation of OS progression and suggesting potential therapeutic targets.

## Introduction

Osteosarcoma (OS), a highly malignant tumor that most commonly originates from bones, is characterized by rapid growth and a high rate of distant metastasis. It is highly malignant and has the capacity to rapidly invade surrounding tissues and undergo distant metastasis via the bloodstream. The lungs are the most common site of metastasis [1]. Over the past several decades, although remarkable advancements have been made in the treatment of osteosarcoma, especially the application of a combination of neoadjuvant chemotherapy and surgery, which has significantly enhanced the survival rate of patients, the five-year survival rate of osteosarcoma patients still hovers at approximately 68%, and 20%-30% of cases are metastatic or recurrent [2]. The poor overall survival of osteosarcoma patients is attributed to their drug resistance and the characteristic of early hematogenous dissemination in inoperable cases. Currently, therapeutic approaches frequently encounter the bottlenecks of chemotherapy resistance and tumor immune escape, which to a large extent are caused by the influence of stem cells and the microenvironment surrounding the tumor [3]. In the tumor microenvironment, mesenchymal stem cells (MSCs) secrete various growth factors, cytokines, and Extracellular vesicles (EVs), interact with tumor cells, and influence the growth, invasion, and immune regulation of the tumor [4]. Therefore, an in-depth investigation of the communication between osteosarcoma-originating stem cells and osteosarcoma is of paramount importance for the research on the mechanism of osteosarcoma occurrence and development.

EVs are a type of nanoparticles encapsulated by a membrane structure and are rich in biological macromolecules, including proteins, mRNA, and non-coding RNA. These molecules can mediate intercellular communication in the tumor microenvironment, thereby affecting the malignant properties of tumor cells, such as proliferation, invasion, and metastasis [5]. In osteosarcoma, bone mesenchymal stem cells (BMSCs) are recruited to the tumor site and communicate with tumor cells by secreting multiple growth factors, cytokines, and EVs, influencing the tumor’s growth, invasion, and immune modulation [6]. EVs play an important role in facilitating intercellular communication between parent cells and recipient cells [7]. Numerous studies have indicated that EVs secreted by bone marrow-derived mesenchymal stem cells (BMSCs) possess inherent targeting specificity for bone tissue, and they can facilitate the proliferation, drug resistance, invasion and metastasis of osteosarcoma cells [8].

Circular RNAs (circRNAs), as a type of non-coding RNA, play a crucial regulatory role in the development and progression of tumors [9]. CircRNAs are less prone to degradation by RNase in the extracellular space, which makes them relatively abundant in EVs and allows them to transmit information over longer distances, affecting the progression of tumors. Existing studies suggest that circular RNAs can exert biological effects through the following manners: circRNAs localized in the cytoplasm can adsorb miRNAs through the molecular sponge function, inhibit the binding between miRNAs and target genes, thereby mediating alterations in downstream signaling pathways and molecular biological functions [10]. CircRNAs that translocate to the nucleus may interact with transcription factors or bind to transcription-related regulatory proteins, thereby influencing the transcriptional regulation of target genes [11]. During the splicing of precursor mRNA, circRNAs can be selectively spliced into circRNAs or linear mRNAs [12]. Currently, the role mechanism of EVs and circRNAs in tumors is still relatively limited. With the in-depth exploration of the expression profile of circRNAs, researchers have gradually revealed their complex regulatory network in tumors. Therefore, further exploration of the circRNAs in EVs and their regulatory mechanisms is expected to provide a new breakthrough point for tumor treatment.

**Scheme 1:**
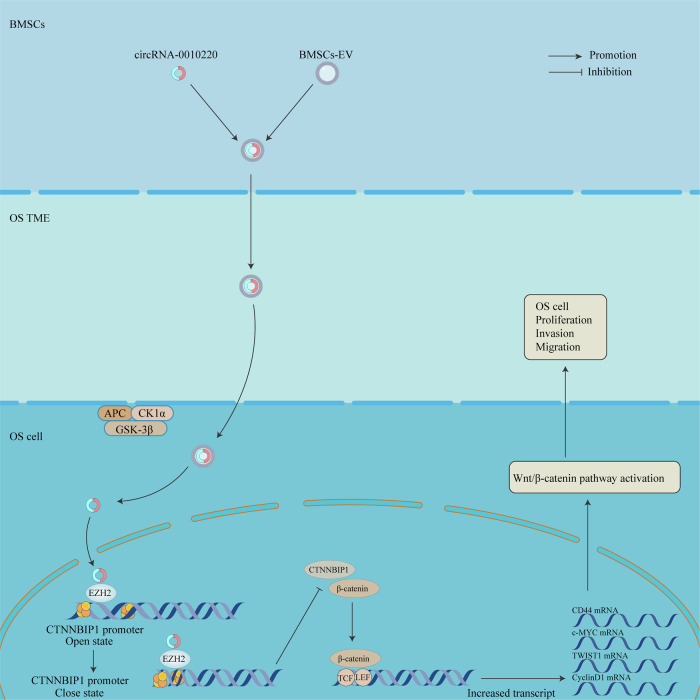
The schematic diagram of circRNA-001022 facilitating the proliferation and metastasis of osteosarcoma via activating the Wnt/β-catenin signaling pathway.

In this study, we demonstrated that BMSCs enhance the proliferation, migration, and invasion of osteosarcoma cells through the delivery of EVs. Subsequently, EVs derived from BMSCs were isolated and subjected to high-throughput sequencing and differential expression analysis, which identified circRNA-0010220. Further investigation revealed that circRNA-001022 recruits histone methyltransferases to induce specific epigenetic modifications in target genes, leading to the activation of the Wnt/β-catenin signaling pathway. This mechanism ultimately promotes the proliferation and metastasis of osteosarcoma (Scheme 1).

## Materials and methods

### Chemicals and reagents

The following antibodies were obtained from Proteintech (Wuhan, Hubei, China): anti-CD63 (#67605-1-Ig, 1:5000), anti-CD9 (#60232-1-Ig, 1:5000), anti-TSG101 (#84334-4-RR, 1:5000), anti-HSP90 (#60318-1-Ig, 1:5000), anti-β-catenin (#66379-1-Ig, 1:5000), and anti-EZH2 (#66476-1-Ig, 1:5000). The Cell Counting Kit-8 was sourced from Dojindo Laboratories (Kumamoto, Japan). PKH26 Exosome Labeling Kit, EDU Assay Kit, ChIP Assay Kit, RNA Immunoprecipitation Assay Kit, and Coomassie Brilliant Blue Rapid Staining Solution were all purchased from Beyotime (Haimen, Jiangsu, China). Lipofectamine™ 2000 (#12566-014) was procured from Invitrogen (USA), and the RNA in situ hybridization kit (#QVC0001) was acquired from Thermo Fisher Scientific. The shRNA plasmids targeting circ-0010220 (sequence: ACATCTGGACTTGGAGTGT) and EZH2 (sequence: GCTGAAGCCTCAATGTTTA) were designed and synthesized by GUANGZHOU RIBOBIO CO., LTD.

### Cell lines and cell culture

Human osteosarcoma cell lines MG-63 and HOS-2 were obtained from the Shanghai Institute of Biochemistry and Cell Biology (SIBCB). The osteosarcoma cells were cultured in RPMI-1640 medium supplemented with 10% Fetal Bovine Serum (FBS), 100 U/mL penicillin, and 100 μg/mL streptomycin. BMSCs were extracted from the medullary cavity of the femurs of 10 patients and cultured in low-glucose DMEM medium, which was supplemented with 20% FBS, 100 U/mL penicillin, and 100 μg/mL streptomycin.

### Isolation and identification of EVs derived from BMSCs

To isolate and purify EVs derived from BMSCs, the culture supernatant of BMSCs cultured for 72 h was collected and centrifuged at 4 °C, 400 g for 8 min, then at 30,000 g for 8 min to obtain the supernatant. The supernatant was then centrifuged at 100,000 g for 70 min to collect the product. The EVs precipitate was stained with 1% uranyl acetate (pH 4.0) and observed under the electron microscope to examine the morphology of EVs. The expression of specific biomarkers for EVs was analyzed by western blot: CD63, CD9, tumor susceptibility gene 101 (TSG101), and heat shock protein 90 (HSP90).

### Cell counting kit-8 assay

The Cell Counting Kit-8 (CCK-8) assay was employed to assess the proliferation of Osteosarcoma cells. Briefly, Osteosarcoma cells with different pretreatments were inoculated in 96-well plates at a density of 8 × 10^3^ cells per well and further cultured for 24 h. Subsequently, 200 μL of CCK8 reaction solution was added to each well, and three blank wells were set for zero adjustment. After incubation in the dark for 2 h, absorbance at 450 nm was measured using a spectrophotometer.

### Colony formation assay

Osteosarcoma cells with different pretreatments were inoculated in 6 cm culture dishes at a density of 500 cells per dish. After seven to ten days of culturing, the cells were fixed with 4% paraformaldehyde solution, stained with 0.1% crystal violet solution, rinsed with clear water for 2-3 times, and then dried in an oven. Photos of the cells in each culture dish were taken and saved, and the number of cell clone formations in each group was statistically analyzed.

### Transwell assay

For the transwell invasion assay, 100 μL of diluted Matrigel was added to the upper chamber of the transwell and incubated in a 37 °C incubator for approximately 1 h. For the Transwell migration assay, no Matrigel was added to the upper chamber. The pretreated osteosarcoma cells were resuspended in serum-free medium and seeded at 4 × 10^4^ cells in the upper chamber of the Transwell, while 600 μL of complete medium with serum was added to the lower chamber. After 24 h of culturing, the medium and matrix gel in the chambers were aspirated, and the remaining matrix gel and cells in the upper chamber were gently wiped off with a cotton swab. The Transwell chambers were fixed with 4% paraformaldehyde and stained with crystal violet, and then observed and counted using an Olympus inverted microscope.

### Wound healing assay

Cells were seeded at a density of 2 × 10^5^ cells per well uniformly in 6-well plates and continuously cultured until the cell density reached approximately 95%. The scratch treatment was initiated by drawing three parallel lines vertically on the cell surface using a 200 μL pipette tip. The cells floating within the scratch area were washed 2-3 times with PBS buffer, serum-free medium was added, and the 6-well plates were placed under a microscope to acquire the 0-h cell scratch images. Five images of different magnifications were randomly selected from each field of view and saved. After 24 h of continuous culture, the 24-h cell scratch images were collected.

### EDU assay

A glass slide was placed in each well of the 6-well plate. 5000 cells were seeded in each well and culture medium was added. After the cells adhered to the wall, 1 mL of culture medium and 1 mL of 2×EDU working solution were added and incubated for 2 h. The culture medium was discarded, and then 1 ml of 4% paraformaldehyde was added to each well for fixation for 15 min. After fixation, each well was washed three times with 1 mL of PBS containing 3% BSA for 5 min each time. 1 mL of PBS containing 3% Triton X-100 was added and incubated at room temperature for 10 min. Each well was washed three times with 1 mL of PBS containing 3% BSA for 5 min each time. PBS was discarded, and 0.5 mL of click reaction solution was added to each well and incubated for 30 min. The click reaction solution was discarded and washed three times with PBS. 10 μL of DAPI was added and incubated in the dark for 10 min. After discarding DAPI and washing, a drop of anti-fluorescence quenching agent was added for sealing, and fluorescence microscopy was used for photography.

### Uptake tracer detection of EVs

Collect 200 μL of the PBS-EVs suspension. According to the instructions of the PKH26 fluorescent cell linker kit, add 400 μL of diluent C solution and mix well. Transfer the mixture to an EP tube, and add 4 μL of PKH26 and 600 μL of diluent C solution into it. Wrap the EP tube with tin foil, shake well and incubate for 5 min. Add 1 mL of 10% FBS to terminate the staining, and then add 6 mL of sterile PBS. Centrifuge at 100,000 g for 70 min. Resuspend the light yellow precipitate in PBS, then transfer it to a 1.5 mL EP tube and store it at –80 °C for future use. Take MG63 and HOS cells in the exponential growth phase, then replace the fresh medium with PKH26-labeled EVs and incubate them in the incubator overnight. Wash with PBS three times, fix MG63 and HOS cells with 4% paraformaldehyde for 15 min, stain the nuclei with DAPI, and observe the EVs uptake under a confocal fluorescence microscope.

### CircRNA sequencing and identification

The extraction of RNA from EVs was conducted using the RA808A-1 kit. Subsequently, rRNA was removed by the MGIEasy kit, and linear RNA was eliminated using the RNase R enzyme to ensure that the samples were rich in circRNAs. The size distribution of RNA was examined using the Agilent Bioanalyzer 2100, and only high-quality samples with RIN values greater than 7 were utilized. The remaining RNA was fragmented into small RNA fragments using the fragmentation buffer, and the small RNA fragments were transcribed into cDNA via a reverse transcription kit. Subsequently, a cDNA library with an average length of 250 ± 50 bp was constructed, and high-throughput sequencing was carried out on the Illumina Novaseq 6000 platform. The acquired clean reads were subjected to standardization processing, and differential expression analysis of circRNAs was performed using the EdgeR package in the R software. circRNAs with significantly differential expression were screened with the criteria of |LogFC|≥2.0 and *P* < 0.05.

### Bioinformatics analysis

For the functional enrichment analysis of the differential gene set from RNA-seq, we initially downloaded the c5.go.bp.v7.4.symbols.gmt subset from MolecularSignaturesDatabase (http://www.gseamsigdb.org/gsea/downloads.jsp) as the background. Genes were mapped to the background set, and enrichment and functional analysis were conducted using the R package clusterProfiler (version 3.14.3) to obtain the results of gene set enrichment. The minimum gene set was set at 5, the maximum gene set at 5000, and the thresholds were set as P < 0.05 and FDR < 0.25.

### Construction and transfection of plasmids

Lipofectamine 2000 lipidosome transfection reagent and plasmid DNA were prepared. When the cells were completely adherent and in a favorable growth condition, the culture medium was replaced with fresh serum-free medium. 4–6 μg of plasmid DNA was mixed in 250 μL of serum-free medium and left to stand at room temperature for 5 min. At the same time, 5–10 μL of Lipofectamine 2000 transfection reagent was mixed in 250 μL of serum-free medium and also left to stand for 5 min. The diluted Lipofectamine 2000 transfection reagent was added to the diluted plasmid DNA and thoroughly mixed, then left to stand at room temperature for 20 min. Subsequently, the mixture was added to the culture dish and co-cultured with the cells for 6–8 h. The culture medium was then replaced with fresh serum-containing medium for continuous culture for 24–48 h. The transfection efficiency was detected by Western blotting after protein extraction.

### Quantitative reverse transcription polymerase chain reaction (RT-qPCR)

RNA was isolated from cultured cells using TRIzol reagent, and its concentration was quantified using a NanoDrop spectrophotometer. First-strand cDNA synthesis was carried out with the PrimeScript™ RT Reagent Kit (Thermo Fisher Scientific, Waltham, MA) according to the manufacturer’s instructions. Quantitative real-time PCR (qPCR) analysis was then performed using the SYBR Green-based qPCR kit (Sangon Biotech, Wuhan, China) on a StepOnePlus Real-Time PCR System (Applied Biosystems). The sequences of the primers used in RT-qPCR are detailed in Table 1 of the supplementary file.

### Subcutaneous tumor formation model

Prepare 6–8-week-old immunodeficient BALB/c nude mice. Digest the cells with trypsin, count them, and resuspend in physiological saline. Select the needle insertion site (typically the axilla or inguinal region, which are areas rich in blood vessels), wipe the needle insertion site with an alcohol swab, advance the needle, and move the needle from side to side before injection. Inject slowly, typically with a volume of 100–150 μL. After the injection is completed, withdraw the needle slowly. The inoculation site should be as far as possible from the needle entry point to prevent leakage and contamination. Weigh the nude mice and measure the tumor size weekly. After 8 weeks, inject 100 μL L of luciferase for in vivo imaging, and after the imaging is completed, euthanize the mice under anesthesia, remove the tumors, and weigh and measure the tumor size.

### Pulmonary metastasis model

6–8-week-old nude mice were selected and injected via the tail vein with 100 μL (3 × 10^6^) osteosarcoma cell suspension for each mouse. 2–4 weeks later, the mice were observed, and the growth and metastasis of the tumor were monitored. The weight, food intake and activity of the nude mice were recorded every three days. At the 8th week after inoculation, the nude mice were sacrificed, and the lungs of the nude mice were removed. The number and volume of the surface nodules of the lungs were calculated, fixed with paraformaldehyde, embedded in low-temperature paraffin, and continuous tissue sections were made. HE staining was performed and observed under an optical microscope to determine the metastatic foci in the lungs.

### Fluorescence in situ hybridization (FISH)

Osteosarcoma cells were fixed with 4% formaldehyde solution at room temperature for 15 min. After washing twice with PBS, 0.5% Triton X-100 was added to treat the osteosarcoma cells for 10 min. RNase A (100 μg/mL) was added and incubated in a cell incubator for 30 min. After washing, 2 mL of pre-hybridization solution was added and incubated at 45 °C for 3 h for pre-hybridization. The probe was added to the pre-hybridization solution and hybridized at 45 °C for 12 h. Add 2x SSC solution at room temperature for washing twice, 5 min each time. Then add 0.1x SSC solution for washing twice, 5 min each time, and then wash twice with 0.1x SSC solution at 45 °C, 15 min each time. DAPI (1 μg/mL) was added for nuclear staining for 8 min. Observation and image acquisition were performed using a fluorescence microscope.

### RNA pull-down

A total of 3 μg of biotin-labeled circRNA-0010220 was supplemented with 100 μL of RNA structure buffer, then denatured at 90 °C for 5 min and placed on ice for 2 min. Subsequently, they were transferred to room temperature for 20 min. Streptavidin beads washed with 60 μL of cell lysis buffer were pre-incubated at room temperature for 1 h. Then, the biotin-labeled circRNA-0010220 and cell lysis buffer were incubated at room temperature for 1 h. 60 μL of streptavidin beads washed with cell lysis buffer were added to the incubation mixture and incubated at room temperature for 1 h. The beads were centrifuged at 3000 rpm at 4 °C for 1 minute. After discarding the supernatant, the beads were washed twice with 1 mL of low-salt wash buffer for 10 min each time and then twice with high-salt wash buffer for 10 min each time. The expression of interacting proteins was detected using Western blot analysis.

### Chromatin Immunoprecipitation (ChIP)

The cells were cross-linked with 1% formaldehyde and blocked with glycine, then washed and digested with Micrococcal nuclease. The chromatin precipitate was suspended in ChIP buffer and sonicated. Aliquots of the fractionated chromatin samples were retained as input controls. The remaining chromatin was incubated with anti-Gli1 antibody. Rabbit immunoglobulin G (IgG) was used as a control. The chromatin was then eluted from the immunoprecipitated chromatin using ChIP wash buffer. The released DNA fragments were then released by incubating at 65 °C with RNase A, followed by proteinase K. The released DNA fragments were purified by column chromatography and amplified by site-specific primers using real-time quantitative PCR.

### RNA-binding protein immunoprecipitation assay

Osteosarcoma cells were washed twice with PBS. After the addition of cell lysis buffer (containing protease inhibitors), the cells were lysed on ice for 30 min. Then they were centrifuged at 14,000 × g at 4 °C for 15 min. Antibodies against EZH2 were added (normal mouse IgG was added in the control group), and the mixture was incubated overnight at 4 °C. Pretreated agarose beads of protein A were added to the mixture and incubated at 4 °C for 4 h to couple the antibody with protein A. After immunoprecipitation, centrifugation was performed at 4 °C for 3 min, and then the supernatant was discarded. The beads were washed three times with lysis buffer. Subsequently, SDS loading buffer was added, and the expression of circRNA-001022 in the RNA-protein complex was detected by reverse transcription PCR and agarose gel electrophoresis.

### Dual-luciferase assay

The culture medium was removed from the culture plate, and then the cells were washed with PBS to eliminate the residual culture medium. An appropriate amount of frozen lysis buffer was added to fully lyse the cells and release the internal enzymes. The culture plate was placed on a shaking platform and shaken at room temperature for 10 min to ensure complete lysis. The lysate was transferred to a centrifuge tube and subjected to rapid centrifugation to remove cell debris and nuclei. The reaction mixture containing the dual luciferase substrate was added to the 96-well plate. The lysate was added to each well to ensure homogeneous mixing. The fluorescence signals of the dual luciferase were read using a photometer. The fluorescence readings were analyzed, and the dual luciferase activity of each well was calculated based on the experimental design and the pre-determined standard curve. The experimental group was compared with the control group, and statistical analysis was conducted to determine the significance of the differences.

### Protein mass spectrometry experiment

The cells are lysed with a mild lysis buffer, and the supernatant is collected. Subsequently, a pulldown experiment for circRNA-0010220 is conducted, and the protein concentration of the precipitated proteins is quantified by the Bradford method. Thereafter, each sample is subjected to a 60-minute reduction treatment at 56 °C in the presence of 10 mM dithiothreitol, and then an alkylation treatment for 60 min at room temperature in the dark using 55 mM iodoacetamide. The alkylated proteins are digested overnight at 37 °C with 1 μg/μl trypsin to generate protein peptide segments. After freeze-drying of the protein peptide segments, they are re-dissolved in 0.5 M ammonium bicarbonate. Each iTRAQ reagent is dissolved in isopropanol and added to the protein peptide segment solution, which is then incubated at room temperature for 2 h and subjected to vacuum drying. The peptide segments are desalted using a Strata X C18 SPE column (Phenomenex, Torrance, CA, USA), and the determination of peptide sequences is performed using a TripleTOF 6600 mass spectrometer (SCIEX, Framingham, MA, USA). The mass spectrometry data are analyzed using ProteinPilot software (version 5.0; SCIEX) to ensure that the false discovery rate of peptide segments is less than 1%.

### Statistical analysis

Statistical analysis was performed using GraphPad Prism 8.0 software. Data are presented as mean ± standard deviation (*x̄* ± *s*). Student’s *t* test was used for comparison between two independent groups. For more than two groups, analysis of variance (ANOVA) was applied if the variance test conditions were met; otherwise, non-parametric tests were used. A *P* value < 0.05 was considered statistically significant.

## Results

### The co-culture of BMSCs facilitates the proliferation, migration and invasion of OS cells

As illustrated in Supplementary Fig. 1A, MG63 and HOS osteosarcoma cells were co-cultured with BMSCs. The CCK-8 assay was employed to evaluate the proliferation rates of osteosarcoma cells in both the NC and BMSCs groups. The results indicated that, compared to the NC group, the proliferation rates of osteosarcoma cells in the BMSCs group were significantly increased at both 24 h and 48 h (Supplementary Fig. 1B, C). Additionally, the colony formation assay revealed a significant increase in the number of clone spheres formed by osteosarcoma cells in the BMSCs group (Supplementary Fig. 1D, E). Furthermore, EDU assays conducted after 48 h of co-culture demonstrated a significant elevation in the EDU-positive rate of MG63 and HOS cells in the BMSCs group (Supplementary Fig. 1F, G).

Moreover, the wound healing assay results showed that the migration capacity of MG63 and HOS cells in the BMSCs group was significantly enhanced (Supplementary Fig. 1H, I). The Transwell experiment further verified that, when compared with the control group, the migration and invasion capabilities of osteosarcoma cells in BMSCs group were significantly enhanced (Supplementary Fig. 1J–L). Collectively, these findings suggest that co-culture with BMSCs promotes the proliferation, migration, and invasion of osteosarcoma cells.

### The co-culture of EVs of BMSCs facilitates the proliferation, migration and invasion of OS cells

**Fig. 1:**
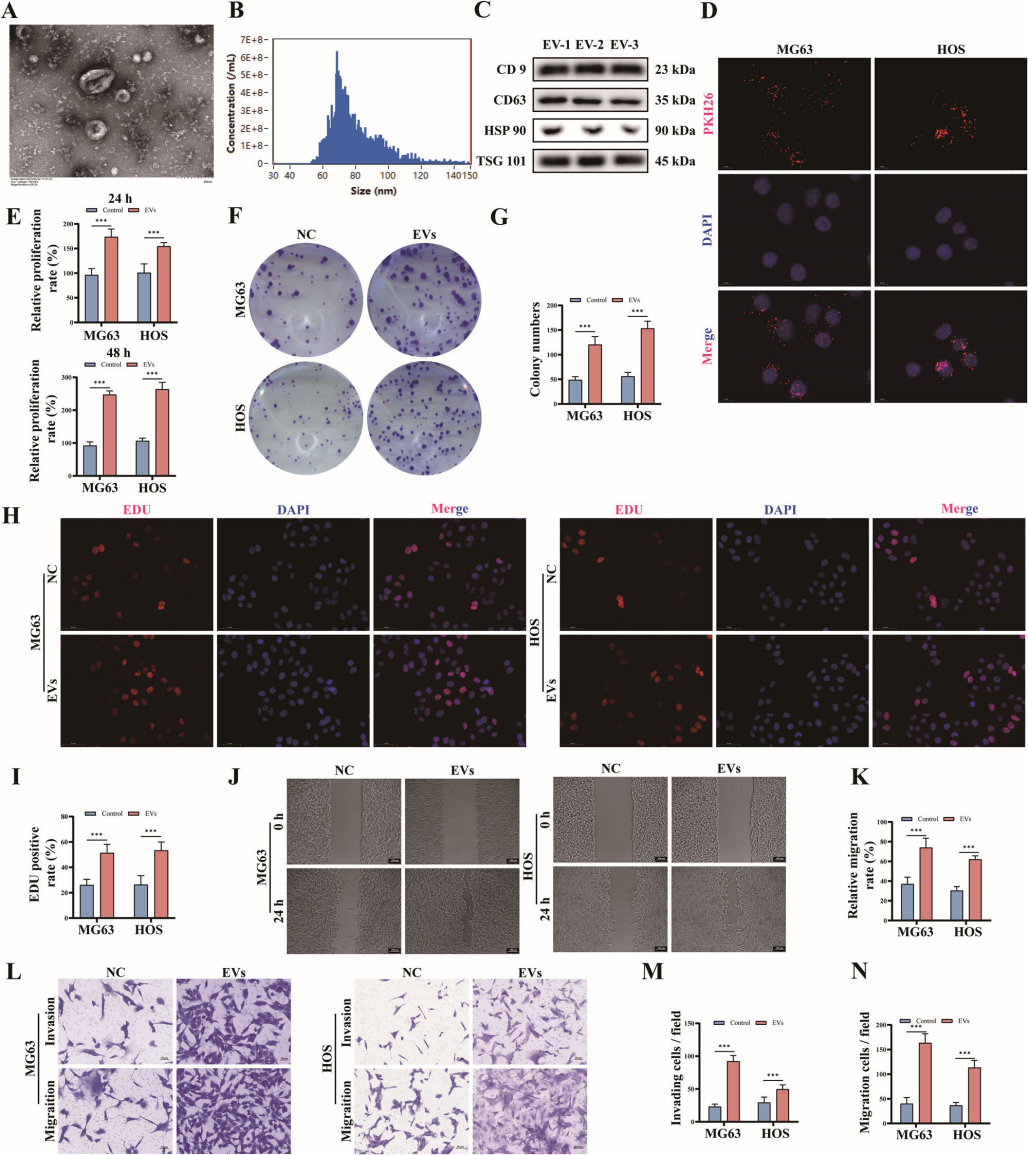
Co-culture of EVs of BMSCs facilitates the proliferation, migration and invasion of OS cells. **A** Transmission electron microscopy of EVs. Scale bar = 200 nm **B** NTA particle size analysis for EVs. **C** Identification of surface markers of EVs using Western blot. **D** EVs tracking experiment: After EVs labeled with PKH26 were co-cultured with osteosarcoma cells for 24 h, observations were made using a confocal microscope. Scale bar = 10 μm. **E** CCK-8 assay after co-culture of OS and EVs for 24 and 48 h. **F**, **G** Cloning formation assay and statistical analysis. **H**, **I** EDU staining and statistical analysis following co-culture of OS and EVs. Scale bar = 50 μm. **J**, **K** Wound healing assay and statistical analysis. Scale bar = 100 μm. **L**–**N** Transwell migration and invasion assays, followed by statistical analysis. Scale bar = 50 μm. ***p < 0.001.

To further investigate whether BMSCs utilize EVs as mediators in intercellular crosstalk, we isolated and characterized EVs secreted by BMSCs. TEM revealed that individual EVs were approximately circular, membrane-encapsulated particles with a characteristic “cup-bottom” morphology (Fig. 1A). The size distribution of the EVs was measured using nanoparticle tracking analysis (NTA), which indicated a diameter range of 30 to 150 nm, with a peak particle size of approximately 70 nm (Fig. 1B). These morphological features are consistent with those of typical extracellular vesicles. Western blot analysis confirmed the presence of key EV markers, including surface proteins CD63 and CD9, as well as intraluminal proteins TSG101 and HSP90, all of which were positive (Fig. 1C). Therefore, the extracted BMSC-derived EVs met the established identification criteria and can be utilized for subsequent experiments.

To investigate the role of EVs in mediating intercellular communication between BMSCs and osteosarcoma cells, a co-culture model was established using Transwell chambers. EVs were able to pass through the pores of the inserts and enter the culture medium in the lower compartment, where they could be internalized by osteosarcoma cells. To enable tracking, EVs derived from BMSCs were labeled with the fluorescent dye PKH26 (Fig. 1D). The results demonstrated that MG63 and HOS osteosarcoma cells efficiently internalized BMSC-derived EVs, suggesting that BMSCs can regulate osteosarcoma cell behavior through EV-mediated signaling. Following co-culture, CCK-8 assays revealed that the proliferation rates of osteosarcoma cells in the EVs group were significantly elevated at both 24 h and 48 h (Fig. 1E). Colony formation assays showed a significant increase in the number of clone spheres formed by osteosarcoma cells in the EVs group (Fig. 1F, G). EDU incorporation assays indicated a significant rise in the EDU-positive rate among osteosarcoma cells exposed to EVs (Fig. 1H, I). These findings collectively suggest that EVs promote the proliferation of osteosarcoma cells. Furthermore, wound healing assays (Fig. 1J, K) and Transwell assays (Fig. 1L–N) demonstrated that co-culture with EVs significantly enhanced the migration and invasion capabilities of osteosarcoma cells. These results indicate that BMSC-derived EVs play a critical role in promoting the proliferation, migration, and invasion of osteosarcoma cells.

### CircRNA-0010220 in EVs derived from BMSCs serves as a crucial molecule facilitating the proliferation, invasion and metastasis of osteosarcoma cells in vitro

**Fig. 2:**
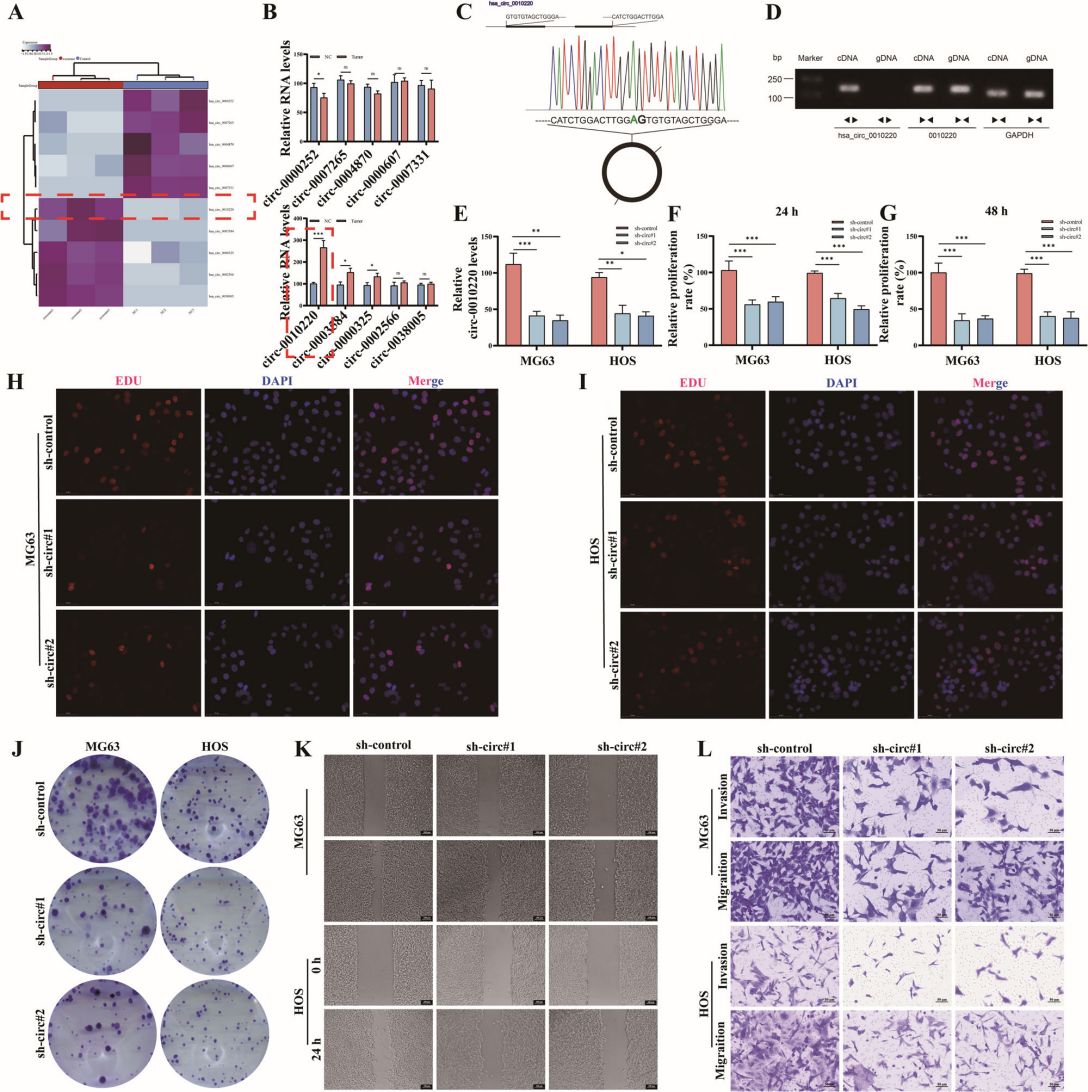
CircRNA-0010220 serves as a crucial molecule facilitating the proliferation, invasion and metastasis of osteosarcoma cells in vitro. **A** Heatmap of the top five upregulated and downregulated circRNAs from RNA-seq. **B** The expression of circRNAs in osteosarcoma was validated using RT-qPCR. **C** Sanger sequencing confirmed that circRNA-0010220 possesses a head-to-tail splicing sequence to form a loop. **D** Agarose gel electrophoresis experiments indicated that the cDNA of circRNA-0010220 was amplifiable outwardly, but gDNA was not. **E** The RT-qPCR experiment validated the transfection efficiency of circRNA-0010220 knockdown. After co-culturing OS cells with EVs derived from BMSCs transfected with circRNA-0010220 knockdown constructs: **F**, **G** CCK-8 assay; **H** Cloning formation assay; **I** Wound healing assay, Scale bar = 100 μm; **J** Transwell migration and invasion assays, Scale bar = 50 μm; **K, L** EDU staining, Scale bar = 50 μm. *p < 0.05; **p < 0.01; ***p < 0.001; ns no significance.

Using RNA-seq technology, we conducted an analysis of the alterations in the circRNA expression profiles of osteosarcoma cells under co-culture conditions with EVs. Based on the criteria of |LogFC|≥ 2.0 and adjusted *P*-value < 0.05, a total of 69 circRNAs with significant differential expression were identified. Among them, 42 circRNAs were upregulated, while 27 were downregulated (Fig. 2A). Specifically, we focused on the five most significantly upregulated circRNAs, namely circRNA-0010220, circRNA-0003584, circRNA-0000325, circRNA-0002566, and circRNA-0038005, as well as the five most significantly downregulated circRNAs, including circRNA-0000252, circRNA-0007265, circRNA-0004870, circRNA-0000607, and circRNA-0007331. RT-qPCR analysis revealed that, among these candidates, circRNA-0010220 exhibited the most pronounced and significant upregulation in osteosarcoma tissues compared to adjacent normal tissues (Fig. 2B). The relevant gene information of circRNA-0010220 was obtained from the circBase database, and its head-to-tail splicing circular sequence was confirmed through Sanger sequencing (Fig. 2C). Agarose gel electrophoresis results demonstrated that the cDNA of circRNA-0010220 could be amplified outwardly, whereas gDNA could not, indicating that circRNA-0010220 exhibits a circular structure (Fig. 2D).

To validate the influence of circRNA-0010220 in BMSC-EVs on the malignant behaviors of osteosarcoma cells, we constructed BMSCs with circRNA-0010220 knockdown via lentiviral transfection. The two interfering plasmids of circRNA-0010220 were respectively named sh-circ#1 and sh-circ#2, while the interfering blank control plasmid was named sh-control. The transfection efficiency of circRNA-0010220 knockdown was verified through RT-qPCR experiments. The results indicated that the stable cell line with circRNA-0010220 knockdown was successfully established (Fig. 2E). Subsequently, the EVs of BMSC after knockdown were collected and co-cultured with osteosarcoma cells for subsequent experiments. The CCK-8 assay results demonstrated that, compared to the sh-control group, the relative proliferation rates of both the sh-circ#1 and sh-circ#2 groups were significantly reduced at 24 h and 48 h (Fig. 2F, G). Further EDU assays revealed that the knockdown of circRNA-0010220 suppressed the in vitro proliferative capacity of osteosarcoma cells (Figs. 2H, I, and S2A). Additionally, knockdown of circRNA-0010220 inhibited the clonal sphere formation ability of osteosarcoma cells (Figs. 2J and S2B). Moreover, wound healing assays (Figs. 2K and S2C) and Transwell assays (Figs. 2L and S2D, E) indicated that the knockdown of circRNA-0010220 repressed the migratory and invasive capabilities of osteosarcoma cells. Collectively, these findings validate that CircRNA-0010220 in EVs derived from BMSCs is a critical molecule facilitating the proliferation, invasion, and metastasis of osteosarcoma cells.

### CircRNA-0010220 in EVs derived from BMSCs is capable of facilitating the proliferation and metastasis of osteosarcoma in vivo

**Fig. 3:**
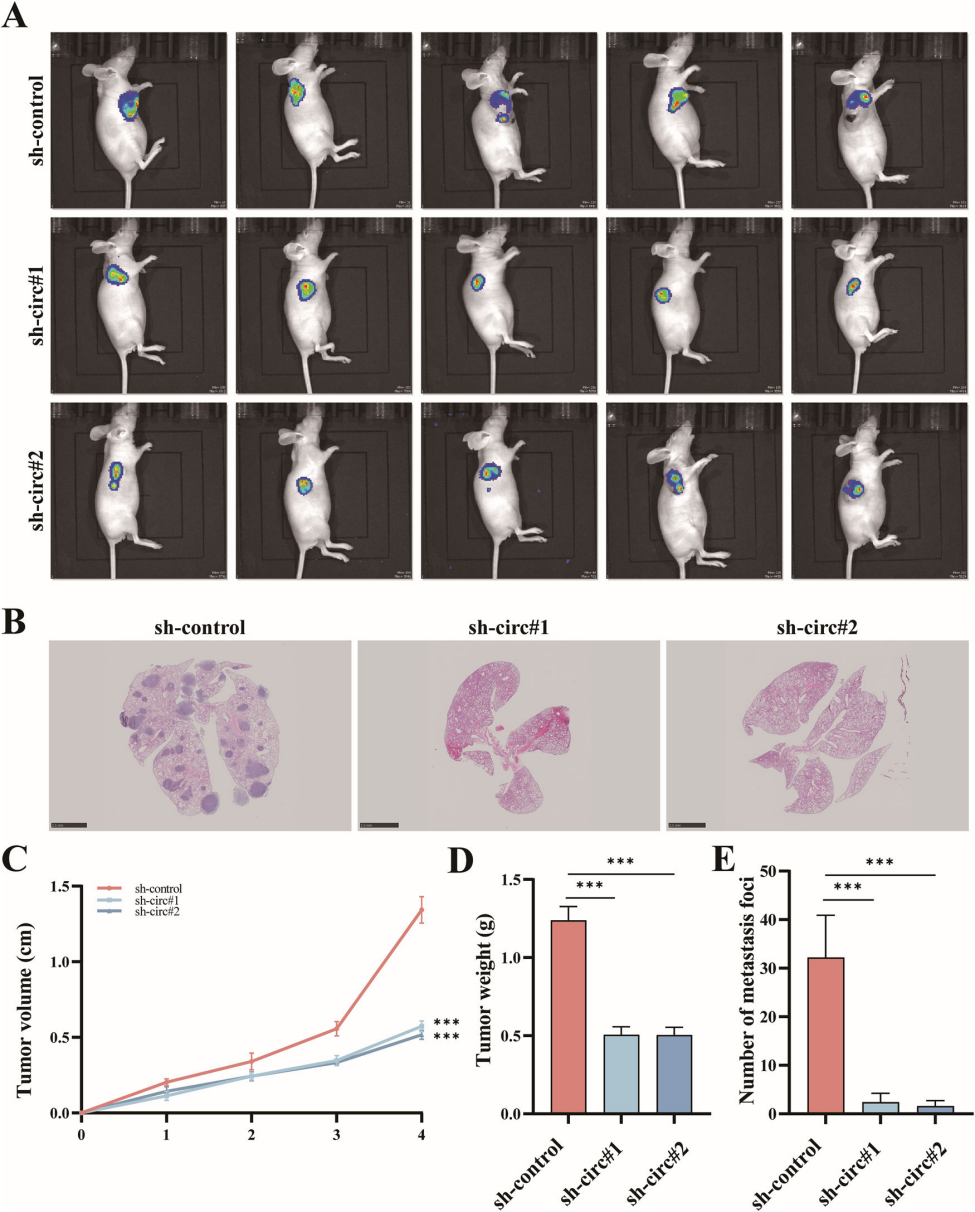
CircRNA-0010220 is capable of facilitating the proliferation and metastasis of osteosarcoma in vivo. **A** Live imaging revealed that the subcutaneous tumorigenic capacity of osteosarcoma cells was significantly decreased following co-culture with EVs originated from BMSCs with circRNA-0010220 knockdown. **B** H&E staining showed that the knockdown of circRNA-0010220 in BMSCs suppresses the in vivo metastasis of osteosarcoma cells. **C**, **D** Statistical analysis of subcutaneous tumor weight and size. **E** Statistical analysis of the number of lung metastatic foci. ***p < 0.001.

To elucidate the influence of circRNA-0010220 on osteosarcoma cells in vivo, we established subcutaneous tumor-bearing nude mouse models using osteosarcoma cells co-cultured with EVs derived from circRNA-0010220 knockdown BMSCs, and conducted in vivo imaging analyses. The results indicated that the size of subcutaneous tumors in nude mice after circRNA-0010220 knockdown decreased significantly (Fig. 3A), and the tumor weight and volume decreased conspicuously (Fig. 3C, D), suggesting that the knockdown of circRNA-0010220 suppressed the proliferation of osteosarcoma cells in vivo. Additionally, by establishing a nude mouse lung metastasis model and performing HE staining on lung metastasis tissues, it was revealed that compared to the sh-control group, the size and number of lung metastatic foci in the sh-circ#1 group and sh-circ#2 group decreased significantly, indicating that the knockdown of circRNA-0010220 inhibited the metastasis of osteosarcoma in vivo (Fig. 3B, E). These results imply that circRNA-0010220 promotes the in vivo invasion and metastasis of osteosarcoma cells.

### CircRNA-0010220 within EVs derived from BMSCs activates the Wnt/β-catenin signaling pathway in OS cells by inhibiting CTNNBIP1

**Fig. 4:**
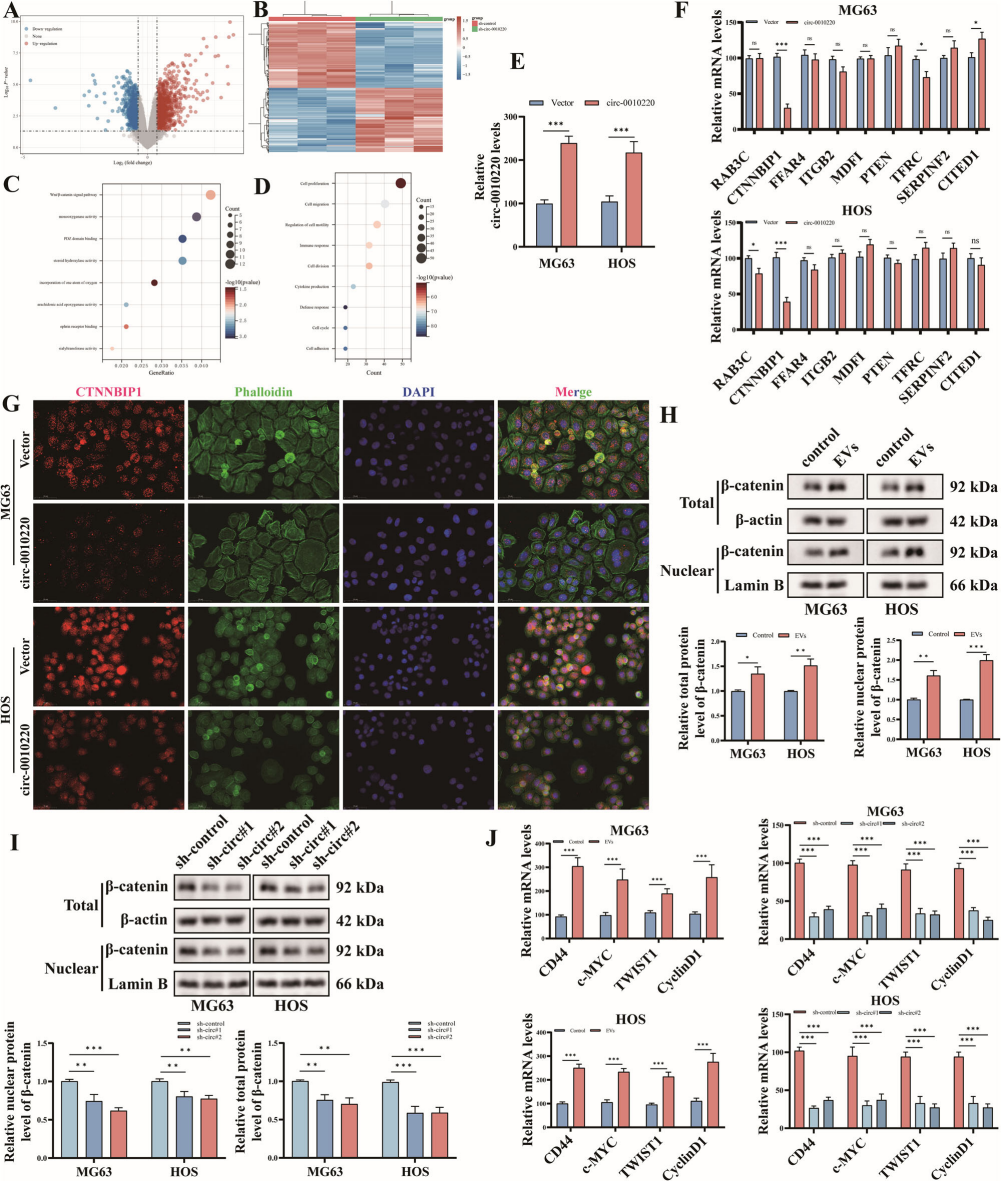
CircRNA-0010220 activates the Wnt/β-catenin signaling pathway in OS cells by inhibiting CTNNBIP1. **A**, **B** Volcano plots and heat maps showed the differential gene sets of RNA-seq. **C** KEGG analysis of the signaling pathways influenced by circRNA-0010220. **D** GO analysis of the biological processes influenced by circRNA-0010220. **E** The RT-qPCR experiment validated the transfection efficiency of circRNA-0010220 overexpression. **F** The RT-qPCR experiment validated the differentially expressed genes enriched in the Wnt/β-catenin pathway. **G** Immunofluorescence staining for CTNNBIP1. Scale bar = 50 μm. **H** Western blot analysis detected the total and nuclear protein levels of β-catenin after co-culture of OS and EVs. **I** Western blot analysis detected the total and nuclear protein levels of β-catenin after co-culture of OS and EVs with circRNA-0010220 knockdown. **J** RT-qPCR detected the expression levels of downstream genes of the Wnt/β-catenin signaling pathway. *p < 0.05; **p < 0.01; ***p < 0.001; ns no significance.

To clarify the mechanism through which circRNA-0010220 within EVs promotes the proliferation and metastasis of osteosarcoma cells, osteosarcoma cells following co-culture were collected for sequencing and differential analysis, uncovering 948 up-regulated and 803 down-regulated differentially expressed genes (Fig. 4A, B). KEGG enrichment analysis suggested enrichment in the Wnt/β-catenin signaling pathway (Fig. 4C). Subsequent GO functional analysis of biological processes demonstrated that these differentially expressed genes mainly influenced cell proliferation and cell migration (Fig. 4D).

Subsequently, we constructed BMSCs with overexpression of circRNA-0010220 and verified its expression level via PCR (Fig. 4E). The overexpression group was designated as the circ-0010220 group, while the blank interference group was named the Vector group. Then EVs from the circ-0010220 group and the Vector group were respectively collected for co-culture with OS cells, and the expression of Wnt/β-catenin signaling pathway-related differential genes in MG63 and HOS cells was detected by RT-qPCR. The results indicated that the expression of CTNNBIP1 in both MG63 and HOS cells of the circ-0010220 group was significantly downregulated (Fig. 4F). Further validation was carried out through immunofluorescence experiments, which also manifested a significant reduction in CTNNBIP1 expression in the circ-0010220 group (Fig. 4G). Subsequently, Western blot was employed to detect the nuclear and total protein levels of β-catenin in osteosarcoma cells co-cultured with EVs. It was found that the nuclear and total protein levels of β-catenin in the EVs group were significantly elevated (Fig. 4H). Additionally, staining of β-catenin and the cytoskeleton by immunofluorescence revealed that the total amount of β-catenin and its expression within the nucleus were significantly increased in MG63 and HOS cells (Fig. S3A). Further detection of the β-catenin protein levels in the sh-circ#1 and sh-circA#2 groups demonstrated that compared with the sh-control group, knockdown of circ-0010220 significantly inhibited the nuclear and total protein levels of β-catenin in osteosarcoma cells (Fig. 4I). Immunofluorescence staining also confirmed this outcome (Fig. S3B).

Furthermore, considering that CTNNBIP1 inhibits the activation of the Wnt/β-catenin pathway by suppressing β-catenin, it exerts no influence on the protein expression of the upstream components of the protein degradation complex, namely APC and GSK-3β. We respectively detected the protein levels of APC and GSK-3β in osteosarcoma cells co-cultured with normal EVs and EVs after circRNA-0010220 knockdown, and found no statistically significant differences (Fig. S3C). Moreover, the expression levels of downstream genes of the Wnt/β-catenin signaling pathway, such as CD44, c-MYC, TWIST1, and Cyclin D1, were examined. The co-culture with the EVs group promoted the activation of the Wnt/β-catenin pathway, while the knockdown of circRNA-0010220 inhibited the activation of this pathway (Fig. 4J). These results suggest that circRNA-0010220 in EVs derived from BMSCs activates the Wnt/β-catenin signaling pathway via CTNNBIP1.

### CircRNA-0010220 in EVs originated from BMSCs recruits EZH2 to facilitate the trimethylation of histone H3K27, thereby suppressing the expression of CTNNBIP1 in the OS

**Fig. 5:**
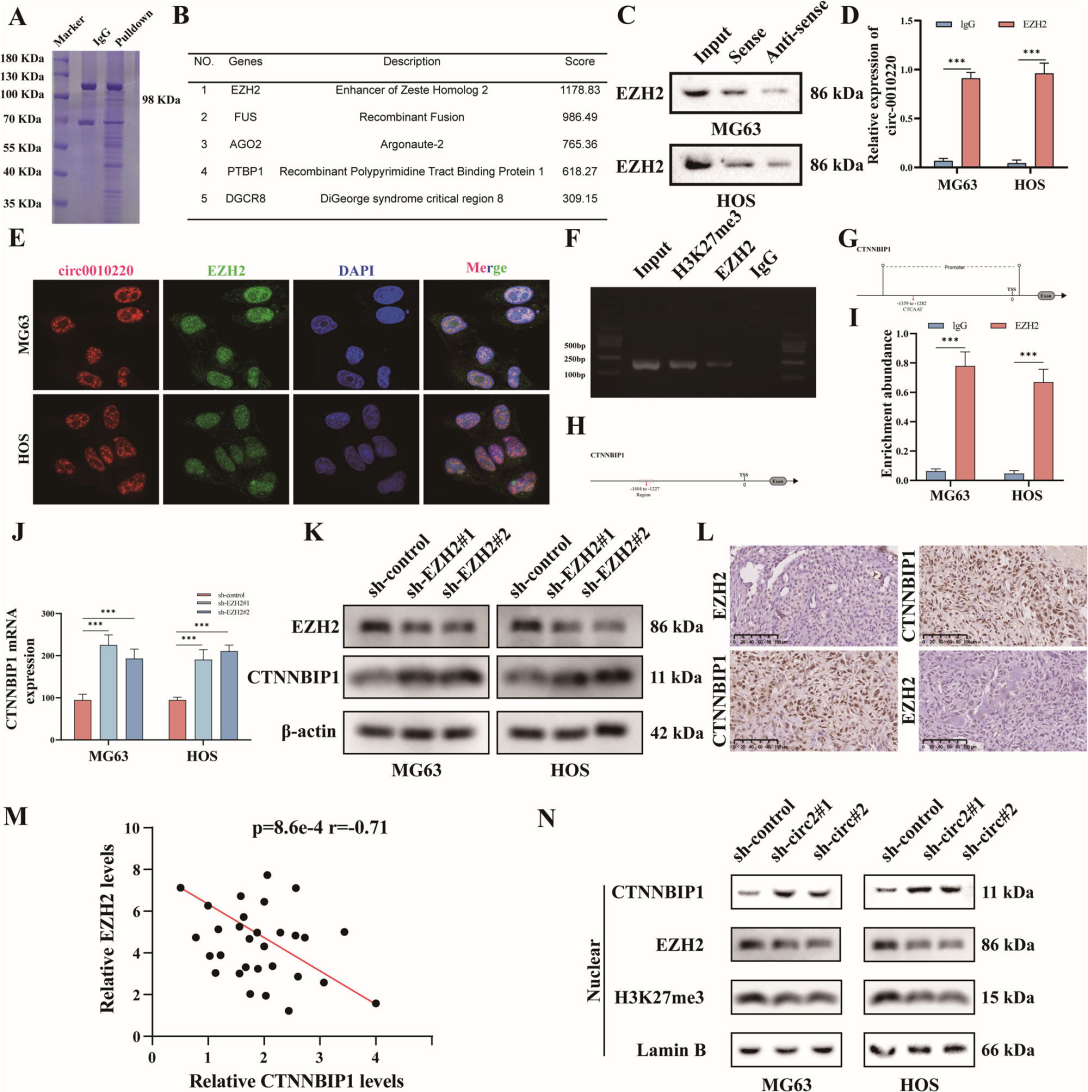
CircRNA-0010220 recruits EZH2 to facilitate the trimethylation of histone H3K27, thereby suppressing the expression of CTNNBIP1 in the OS. **A**, **B** After pulldown assay, the Coomassie Brilliant Blue assay and proteomic analysis were performed to identify the interacting proteins of circRNA-0010220. **C**, **D** Western blot assays and RIP validation indicated that circRNA-0010220 recruited EZH2. **E** The FISH assay validated the co-localization of circRNA-0010220 and EZH2. **F**, **G** The CHIP assay confirmed that EZH2 binds to the H3K27 region of the CTNNBIP1 promoter. **H, I** The dual-luciferase reporter assay confirmed that EZH2 binds to the promoter region of CTNNBIP1. **J, K** The detection of CTNNBIP1 mRNA and protein levels subsequent to EZH2 knockdown. **L**, **M** Immunohistochemical staining of EZH2 and CTNNBIP1 was carried out in osteosarcoma tissues, along with the Pearson correlation analysis. Scale bar = 100 μm. **N** Western blot experiments were performed to validate the influence of circRNA-0010220 on the nuclear protein levels of CTNNBIP1, EZH2, and H3K27me3 in osteosarcoma. ***p < 0.001.

In our previous experiments, it was discovered that circRNA-0010220 in EVs derived from BMSCs activated the Wnt/β-catenin pathway and facilitated the proliferation, invasion, and metastasis of osteosarcoma. To elucidate how circRNA-0010220 participates in the activation of the Wnt/β-catenin signal, we conducted a pull-down assay followed by Coomassie brilliant blue staining and protein spectrum analysis, and identified EZH2 as a potential interacting protein of circRNA-0010220 (Fig. 5A, B). The results of Western blot after pull-down also indicated that circRNA-0010220 enriched the EZH2 protein in osteosarcoma cells (Fig. 5C). Furthermore, RIP experiments using an EZH2 antibody revealed that circRNA-0010220 was an interacting RNA of EZH2 (Fig. 5D). Additionally, FISH experiments verified that circRNA-0010220 and EZH2 were co-localized in the nucleus of osteosarcoma cells (Fig. 5E). These results demonstrated that circRNA-0010220 could recruit EZH2. EZH2, as a histone methyltransferase, binds to histone H3K27 in the promoter region of target genes, resulting in trimethylation of histone H3K27 (H3K27me3), thereby regulating the transcription of target genes. CHIP-PCR was employed to verify the binding region of EZH2 and CTNNBIP1, and the results showed that EZH2 bound to the H3K27 region of the CTNNBIP1 promoter (Fig. 5F, G). Further, dual-luciferase reporter assays confirmed that EZH2 bound to this promoter region of CTNNBIP1 (Fig. 5H, I).

To validate the regulatory effect of EZH2 on CTNNBIP1 expression in osteosarcoma cells, EZH2 was knocked down (sh-EZH2#1 and sh-EZH2#2) in OS cells and co-cultured with EVs derived from BMSCs. Subsequently, the mRNA level (Fig. 5J) and protein level (Fig. 5K) of CTNNBIP1 were examined. The outcomes demonstrated that the knockdown of EZH2 attenuated the inhibitory impact of circRNA-0010220 on the mRNA and protein levels of CTNNBIP1. Further, the expression correlation between EZH2 and CTNNBIP1 in osteosarcoma patient tissues was verified through immunohistochemistry. The results indicated a negative correlation in the expression levels of EZH2 and CTNNBIP1 in osteosarcoma tissues (Fig. 5L). As shown in Fig. 5M, Pearson correlation analysis of 30 osteosarcoma tissue samples revealed a significant negative correlation between EZH2 and CTNNBIP1 expression (*r* = –0.71, *P* < 0.01). After co-culturing osteosarcoma cells with EVs derived from BMSCs with circRNA-0010220 knockdown, the protein levels of CTNNBIP1, EZH2, and H3K27me3 within the cell nucleus were detected. The results demonstrated that in the sh-circ#1 and sh-circ#2 groups, the nuclear protein expression of CTNNBIP1 in the osteosarcoma cells was significantly increased, while the nuclear protein levels of EZH2 and H3K27me3 were markedly reduced (Fig. 5N). The aforementioned results suggested that circRNA-0010220 recruited EZH2 to facilitate the trimethylation of histone H3K27, thereby suppressing the expression of CTNNBIP1 in the OS.

### The inhibition of the Wnt/ β-catenin signaling pathway reversed the promotional effect of circRNA-0010220 on the proliferation, migration and invasion of OS cells

**Fig. 6:**
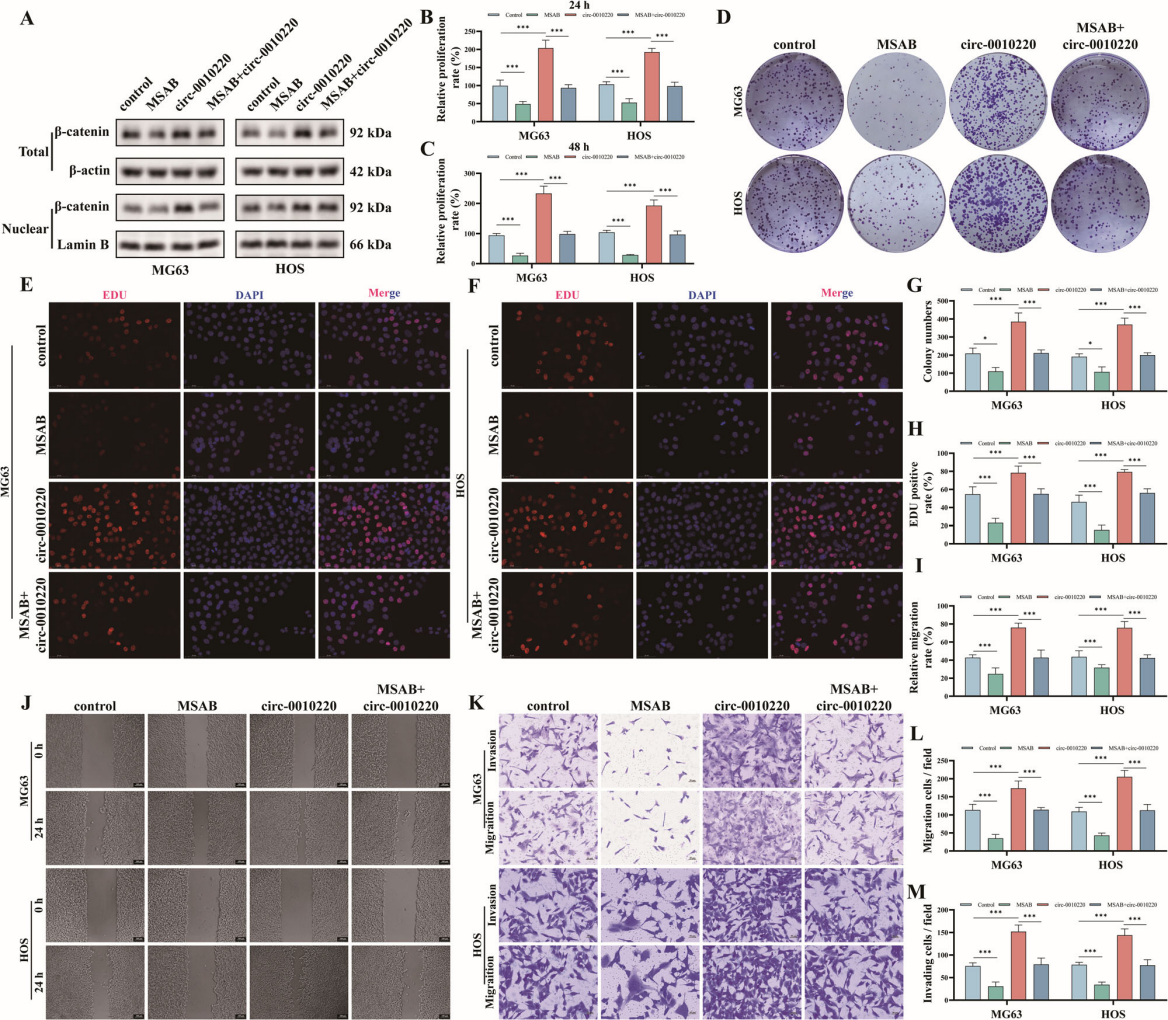
The inhibition of the β-catenin/Wnt signaling pathway reversed the promotional effect of circRNA-0010220 on the proliferation, migration and invasion of OS cells. **A** Western blot was employed to validate the inhibitory effect of the inhibitor MSAB on β-catenin. After co-culturing OS cells with EVs derived from BMSCs transfected with circRNA-0010220 overexpression constructs and treating with MSAB: **B**, **C** CCK-8 assay; **D**, **G** Cloning formation assay and statistical analysis; **E**, **F**, **H** EDU staining and statistical analysis, Scale bar = 50 μm; **J**, **I** Wound healing assay and statistical analysis, Scale bar = 100 μm; **K**–**M** Transwell assays and statistical analysis, Scale bar = 50 μm. ***p < 0.001.

To further ascertain that circRNA-0010220 within EVs exerts its biological effects via the Wnt/β-catenin signaling pathway, we utilized the β-catenin inhibitor MSAB to validate our findings. The Western blot results demonstrated that MSAB effectively suppressed both total and nuclear β-catenin protein levels in both the control group and the circ-0010220-overexpressing group (Fig. 6A). Consistent with these observations, immunofluorescence staining of β-catenin and the cytoskeleton in osteosarcoma cells yielded similar results (Fig. S3A, C). Additionally, the detection of the expression of downstream genes of the β-catenin/Wnt signaling pathway further confirmed the inhibitory effect of MSAB on this pathway (Fig. S3B, D). Subsequently, CCK-8 assays (Fig. 6B, C), colony formation assays (Fig. 6D, G), and EDU staining (Fig. 6E, F, H) were conducted to evaluate the proliferative capacity of MG63 and HOS osteosarcoma cells. These experiments revealed that inhibition of the Wnt/β-catenin signaling pathway significantly attenuated the proliferation-promoting effects of circRNA-0010220. Moreover, wound healing assays (Fig. 6J, I) and Transwell assays (Fig. 6K–M) were performed to assess cell migration and invasion. Consistently, inhibition of the Wnt/β-catenin signaling pathway also mitigated the pro-migratory and pro-invasive effects of circRNA-0010220.

## Discussion

BMSCs exert multifaceted roles in tumor progression. They can impact tumor growth, angiogenesis, metastasis, and tumor microenvironment remodeling by directly secreting bioactive substances. For example, Qi et al. discovered that in gastric cancer, BMSCs facilitate gastric cancer progression and metastasis by secreting thrombospondin-2 [13]. Furthermore, BMSCs can also engage in long-distance extracellular communication through EVs and participate in modulating multiple processes such as tumor proliferation, migration, invasion, and immune escape, thereby playing a crucial role in tumorigenesis and development [14]. For instance, in cholangiocarcinoma, studies have revealed that cancer-related EVs can reduce the quantity of cytokine-induced NK-like T cells, thereby suppressing the anti-tumor activity of the tumor and ultimately facilitating tumor immune escape [15]. As the parental cells of osteosarcoma, the EVs secreted by BMSCs possess a natural targeting specificity for bone tissue, thus exerting more significant effects on the proliferation, drug resistance, invasion, and metastasis of osteosarcoma cells [8]. In this study, through co-culturing osteosarcoma cells HOS and MG63 with BMSCs and the EVs derived therefrom, it was found that the EVs originated from BMSCs could conspicuously promote malignant biological behaviors of osteosarcoma cells, such as proliferation, migration, and invasion.

Owing to its distinctive closed-loop structure, circRNAs demonstrate greater stability in the extracellular milieu compared to other RNA molecules and are capable of effectively resisting degradation by endonucleases outside EVs. Consequently, it plays a more intricate role in the genesis, development, and metastasis of tumors [16]. Through RNA-seq screening of EVs, we identified the potential functional molecule circRNA-0010220 within EVs and validated it through in vivo and in vitro experiments that circRNA-0010220 in EVs derived from BMSCs facilitated the proliferation and metastasis of osteosarcoma cells. Further research indicated that by conducting sequencing and bioinformatics analyses of osteosarcoma cells co-cultured with EVs and subsequent experimental verifications, it was discovered that circRNA-0010220 exerted its biological effects by regulating CTNNBIP1, a crucial inhibitor of the Wnt/β-catenin pathway. CTNNBIP1 is a key inhibitor in the Wnt/β-catenin signaling pathway, which directly binds to β-catenin and impedes its interaction with TCF/LEF transcription factors, thereby suppressing the activity of the Wnt signaling pathway [17]. A plethora of studies have manifested that CTNNBIP1 influences tumor progression by regulating the Wnt/β-catenin signaling pathway. For instance, in glioma, miR-603 activates the Wnt/β-catenin signaling pathway by inhibiting CTNNBIP1, promoting the proliferation of glioma cells [18]. Whereas in hepatocellular carcinoma, LINC01391 inhibits the proliferation and invasion of hepatocellular carcinoma by promoting the expression of CTNNBIP1 and inactivating the Wnt/β-catenin signaling pathway [19]. Our study revealed that circRNA-0010220 promotes the proliferation, migration, and invasion of osteosarcoma cells by inhibiting the expression of CTNNBIP1 and activating the Wnt/β-catenin signaling pathway.

Typically, circRNAs possess distinct mechanisms of action based on their cellular localization, thereby influencing tumor progression. The circRNAs within the cytoplasm primarily act as miRNA sponges to decrease the availability of miRNAs, thereby alleviating the inhibition on downstream mRNA targets. For example, in breast cancer, circRNA-001783 promotes the progression of breast cancer cells by sponging miRNA-200c-3p and releasing the inhibition of miRNA-200c-3p on the target genes ZEB1/2 [20]. Additionally, the circRNAs in the cytoplasm can also regulate tumor progression by influencing cellular signaling pathways. For instance, in gastric cancer, circPDIA4 binds to ERK1/2 and continuously overactivates the MAPK pathway by hindering DUSP6-mediated dephosphorylation of ERK1/2, thereby facilitating gastric cancer progression [21]. The circRNAs within the nucleus can directly impact the regulation of target genes or participate in post-transcriptional modifications of genes. For example, Xu et al. discovered that in pancreatic ductal adenocarcinoma, circRNA-0007919 recruits FOXA1 and TET1, reduces methylation of the LIG1 promoter and enhances its transcription, strengthens DNA damage repair, and leads to gemcitabine resistance [22]. Furthermore, Zheng et al. identified that in renal cell carcinoma, circPPAP2B regulates the interaction between HNRNPC and splicing factors PTBP1 and HNRPK, influencing the alternative splicing of HNRNPC pre-mRNA [23]. In this study, pull-down experiments, Coomassie Brilliant Blue staining, protein mass spectrometry identification, and Western blot verification were performed, and it was revealed that circRNA-0010220 interacts with EZH2. The co-localization of circRNA-0010220 and EZH2 in the nuclei of osteosarcoma cells was verified through FISH experiments.

EZH2 is a histone methyltransferase that catalyzes the trimethylation of lysine at position 27 of histone H3 in the promoter region of target genes, resulting in highly condensed chromatin in this promoter region and thereby inhibiting the expression of target genes [24]. A considerable number of studies have demonstrated that EZH2 is an upstream activator of the Wnt/β-catenin signaling pathway. For example, exosomes derived from mesenchymal stem cells target and inhibit EZH2 through miR-133b, thereby regulating the Wnt/β-catenin pathway [25]. Furthermore, EZH2 can recruit the deubiquitinating enzyme USP7 to enhance the stability of β-catenin and activate the activity of the Wnt/β-catenin pathway [26]. In the present study, we validated through ChIP-PCR, dual-luciferase reporter assays, RT-qPCR, and Western blot that EZH2 acts on the promoter region of CTNNBIP1, inducing trimethylation of histone H3K27 and suppressing the expression of CTNNBIP1. Immunohistochemical experiments further substantiated that the expressions of EZH2 and CTNNBIP1 in tissues of osteosarcoma patients are negatively correlated. Additionally, experiments employing the Wnt/β-catenin signaling pathway inhibitor MSAB once again verified the mechanism of action of circRNA-0010220 in osteosarcoma.

In summary, this study demonstrates that circRNA-0010220 derived from BMSCs promotes the recruitment of EZH2 in osteosarcoma cells, leading to the methylation of the CTNNBIP1 gene and the subsequent inhibition of CTNNBIP1 expression. This, in turn, activates the Wnt/β-catenin signaling pathway, ultimately enhancing the proliferation and metastasis of osteosarcoma. These discoveries enrich the regulatory network of BMSC-derived extracellular vesicles on osteosarcoma proliferation and metastasis and also offer potential therapeutic targets. Despite our recognition that the single subcutaneous metastasis model and the lung metastasis model cannot fully simulate the complex microenvironment during osteosarcoma metastasis and have certain limitations, our conclusion is amply supported by repeated verifications through in vivo and in vitro experiments. Furthermore, whether circRNA-0010220 can influence the biological behavior of osteosarcoma through other mechanisms remains to be further investigated.

## Conclusion

BMSCs deliver circRNA-0010220 to OS cells via EVs. In OS cells, circRNA-0010220 recruits EZH2 and binds to the promoter region of the CTNNBIP1 gene, leading to trimethylation of histone H3K27 in this region. This modification inhibits CTNNBIP1 expression and subsequently activates the Wnt/β-catenin signaling pathway, ultimately promoting the proliferation, invasion, and metastasis of osteosarcoma.

## Supplementary information

Supplementary Materials

Supplementary Materials

## Data Availability

The data that support the findings of this study are available on request from the corresponding author.
