# Supplementary material for: BMSC-derived extracellular vesicles enhance osteosarcoma proliferation and metastasis via the circRNA-0010220/β-catenin pathway
Source: Cell Death Dis. 2026 Mar 25;17(1):376. doi: 10.1038/s41419-026-08655-8 (PMC13039318; doi:10.1038/s41419-026-08655-8)
Supplement: Supplementary file 1 — Supplementary Materials [file 41419_2026_8655_MOESM1_ESM.docx]

**Supplementary Materials**

**BMSC-Derived Extracellular Vesicles Enhance Osteosarcoma Proliferation and Metastasis via the circRNA-0010220/β-Catenin Pathway**

Runsang Pan^1, 3, †^, Yujie Pan^2, 3, †^, Wanyuan Ruan^3, †^, Hao Zheng^3^, Guangfu Jiang^2^, Jianyang Li^1, *^, Xiaobin Tian^2, *^, Li Sun^1, *^

^1^ Department of Orthopedics, GuiZhou Provincial People’s Hospital, Guiyang 550000, GuiZhou Province, China

^2^ Department of Emergency, the affiliated Hospital of Guizhou Medical University, Guiyang 550004, Guizhou Province, China

^3^ School of Clinical Medicine, Guizhou Medical University, Guiyang 550000, Guizhou, China

***Corresponding author**

**Jianyang Li,** Department of Orthopedics, GuiZhou Provincial People’s Hospital, Guiyang 550000, Guizhou Province, China. Email: Lijianyang721@163.com.

**Xiaobin Tian,** School of Clinical Medicine, Guizhou Medical University, Guiyang 550000, Guizhou, China. Email: txb6@vip.163.com.

**Li Sun,** Department of Orthopedics, GuiZhou Provincial People’s Hospital, Guiyang 550000, Guizhou Province, China. Email: [lisun@gzu.edu.cn](mailto:lisun@gzu.edu.cn).

**† These authors contributed equally to this work.**


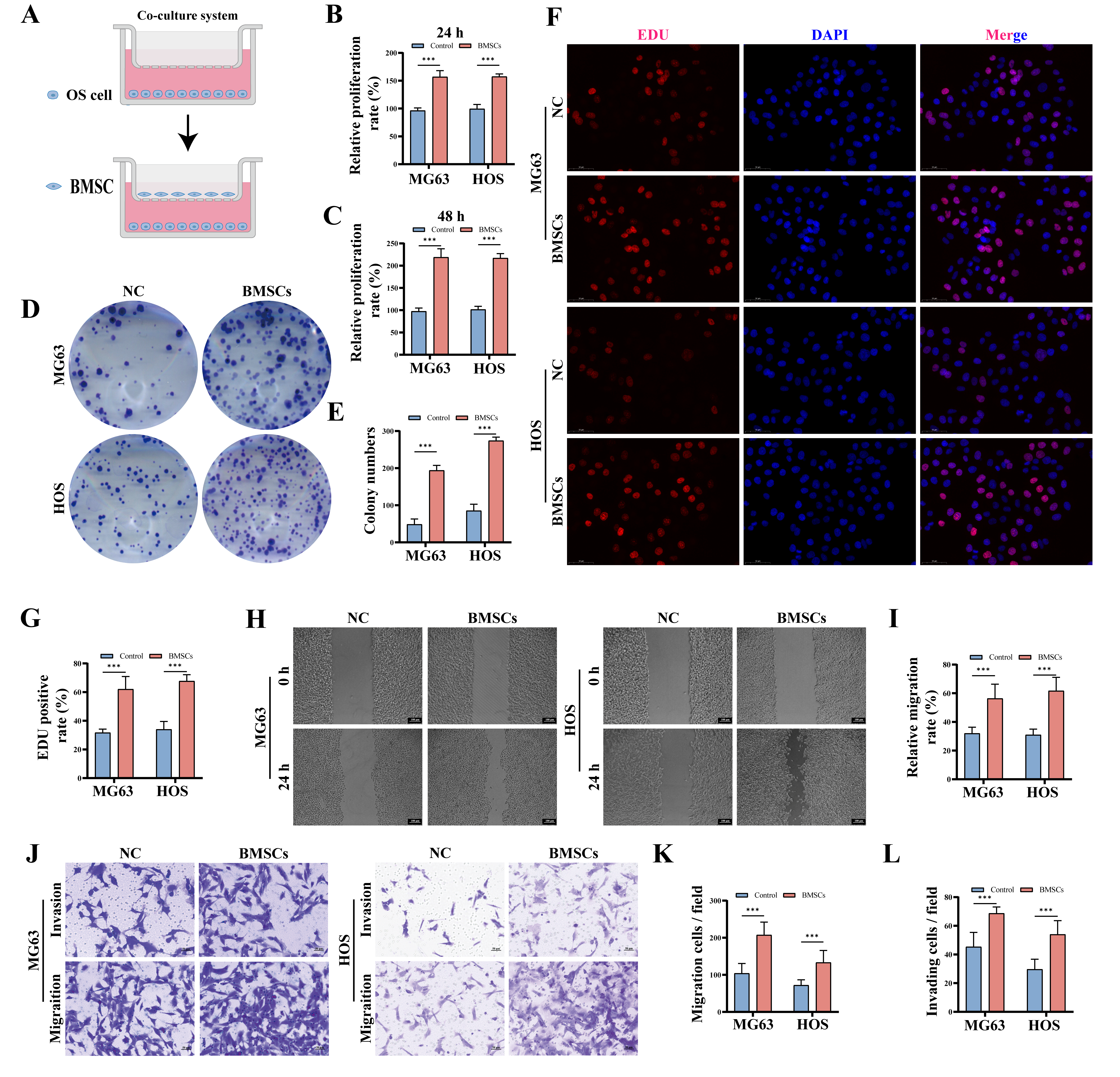


**Figure S1. Co-culture of BMSCs facilitates the proliferation, migration and invasion of OS cells. A)** Schematic diagram of co-culture of osteosarcoma cells and BMSCs. **B, C)** CCK-8 assay after co-culture of OS and BMSCs for 24 and 48 hours. **D, E)** Cloning formation assay and statistical analysis. **F, G)** EDU staining and statistical analysis following co-culture of OS and BMSCs. Scale bar = 50 μm. **H, I)** Wound healing assay and statistical analysis. Scale bar = 100 μm. **J-L)** Transwell migration and invasion assays, followed by statistical analysis. Scale bar = 50 μm. ****p* < 0.001.


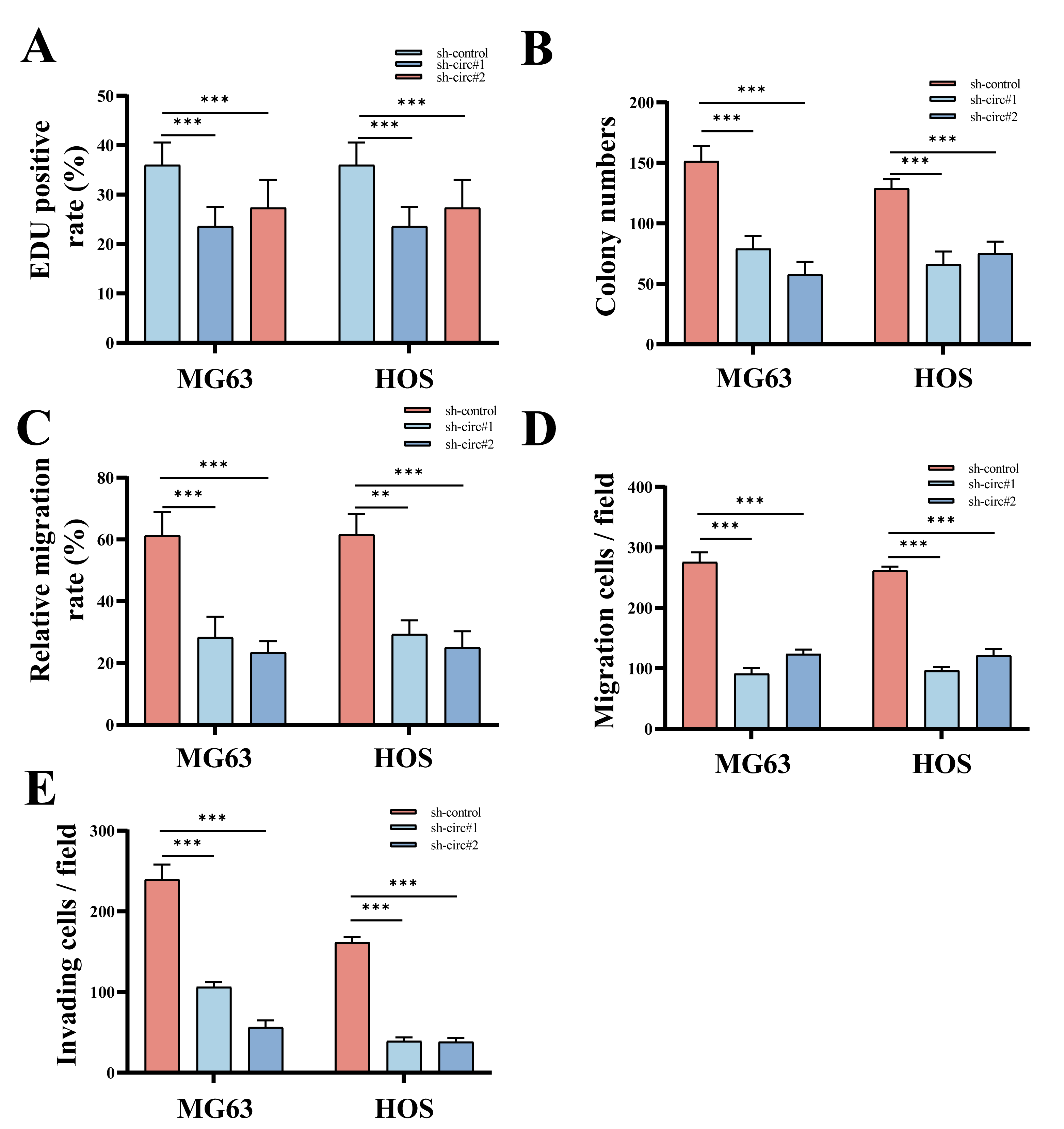


**Figure S2. CircRNA-0010220 serves as a crucial molecule facilitating the proliferation, invasion and metastasis of osteosarcoma cells *in vitro*.** After co-culturing OS cells with EVs derived from BMSCs transfected with circRNA-0010220 knockdown constructs: **A)** Statistical analysis for EDU staining. **B)** Statistical analysis for cloning formation assay; **C)** Statistical analysis for wound healing assay; **D, E)** Statistical analysis for Transwell migration and invasion assays; ****p* < 0.01; ****p* < 0.001.


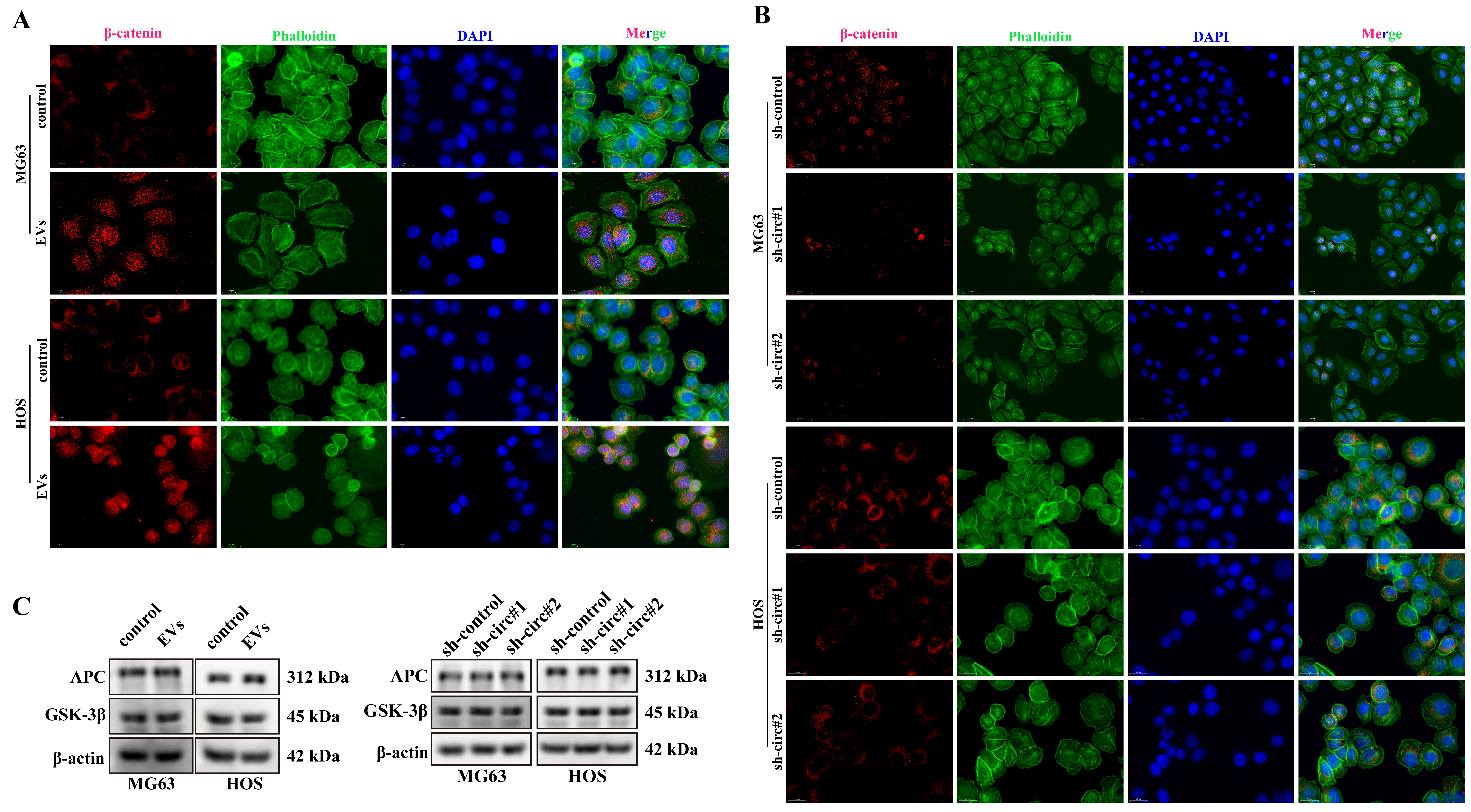


**Figure S3. CircRNA-0010220 activates the Wnt/β-catenin signaling pathway in OS cells by inhibiting CTNNBIP1. A)** Immunofluorescence staining for β-catenin after co-culture of OS and EVs. Scale bar = 50 μm. **B)** Immunofluorescence staining for β-catenin after co-culture of OS and EVs with circRNA-0010220 knockdown. Scale bar = 50 μm.

**Figure S4. The inhibition of the β-catenin/Wnt signaling pathway reversed the promotional effect of circRNA-0010220 on the proliferation, migration and invasion of OS cells.** After co-culturing OS cells with EVs derived from BMSCs transfected with circRNA-0010220 overexpression constructs and treating with MSAB: **A, C)** Immunofluorescence staining for β-catenin. Scale bar = 50 μm. **B, D)** RT-qPCR detected the expression levels of downstream genes of the Wnt/β-catenin signaling pathway. Scale bar = 50 μm. ****p* < 0.001.

**Table 1. The sequences of the primers**

| **Gene** | **Sense primer** | **Antisense primer** |
| --- | --- | --- |
| hsa_circ_0010220 | GCTCTGGGAGATGCTGGAAT | GATCTCCTATGGCAGGCTGTG |
| 0010220-linear | GCGTGACACACATGGTGAAG | AGGAGGAAGGTGGTCCTGGT |
| CTNNBIP1 | TCATGCTGCGGAAGATGG | ATCACCACGTCCTCTGCA |
| CD44 | GCAGGAAGAAGGATGGATAT | CAGAGTAGAAGTTGTTGGATG |
| C-MYC | GGCTCCTGGCAAAAGGTCA | CTGCGTAGTTGTGCTGATGT |
| TWIST1 | GACAAGCTGAGCAAGATTC | ATCCTCCAGACCGAGAAG |
| Cyclin D1 | GAACAGAAGTGCGAGGAG | GCGGTAGTAGGACAGGAA |
| GAPDH | ccacagtccatgccatcactg | gtcaggtccaccactgacacg |
