# Supplementary figures and images for: BMSC-derived extracellular vesicles enhance osteosarcoma proliferation and metastasis via the circRNA-0010220/β-catenin pathway
Source: Cell Death Dis. 2026 Mar 25;17(1):376. doi: 10.1038/s41419-026-08655-8 (PMC13039318; doi:10.1038/s41419-026-08655-8)

Figure 1

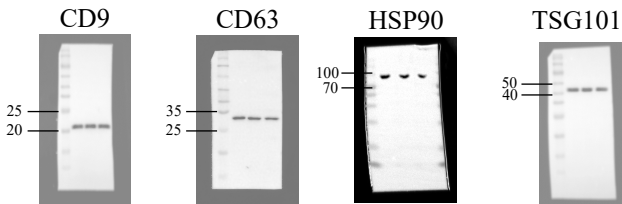

Figure 4

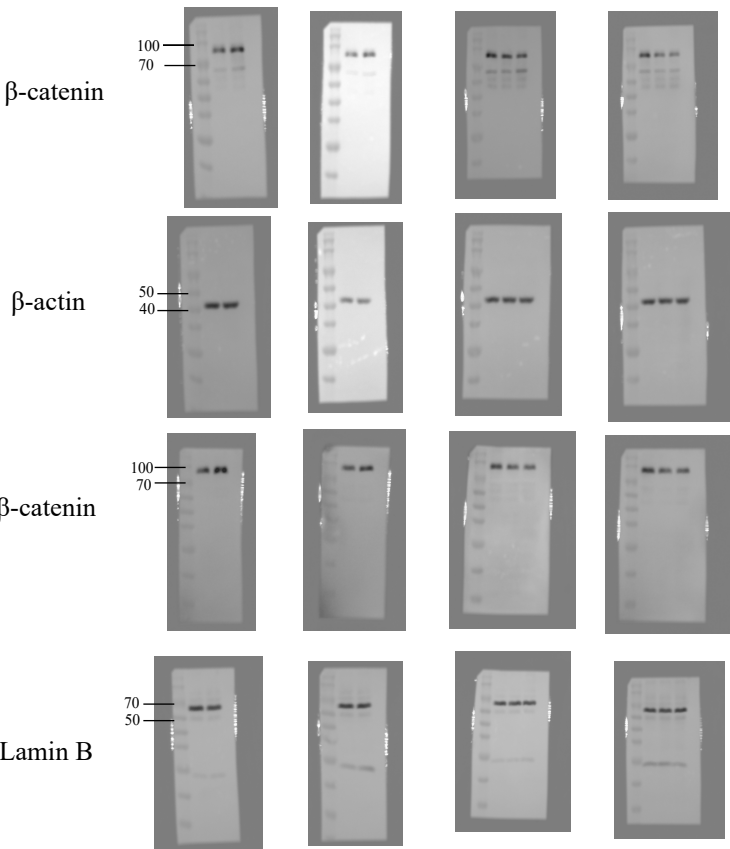

Figure S3

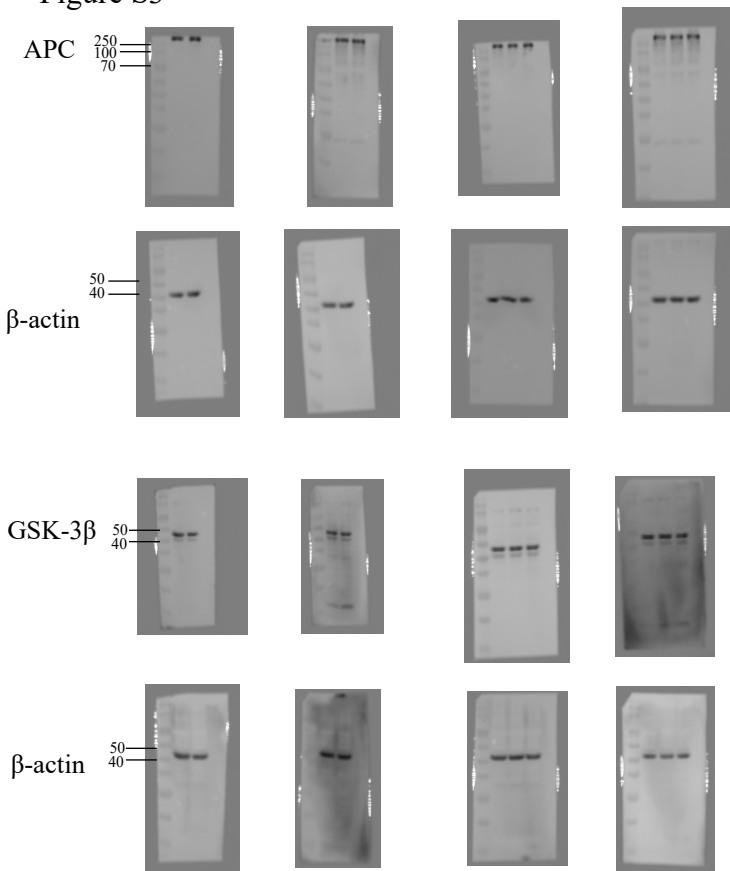

Figure 5C

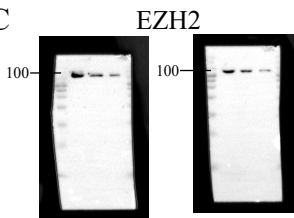

Figure 5K

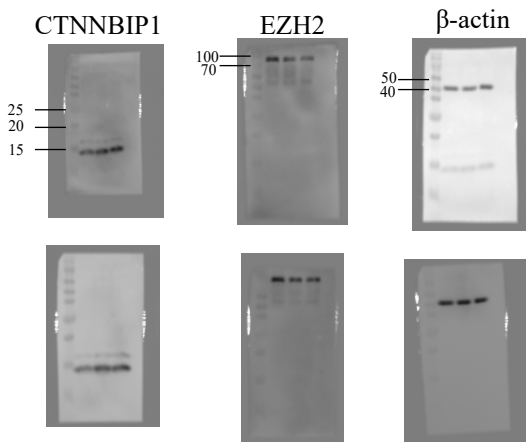

Figure 5N

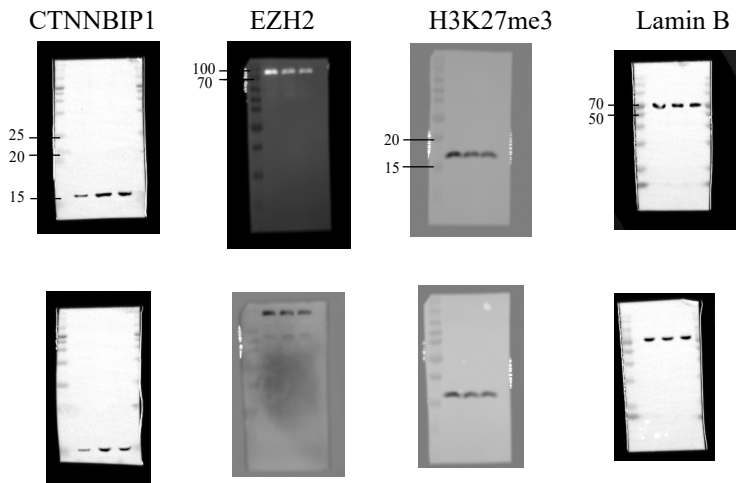

Supplement: Supplementary file 2 — Supplementary Materials [file 41419_2026_8655_MOESM2_ESM.pdf]
